# Prevalence of depression and anxiety in patients requesting physicians’ aid in dying: cross sectional survey

**DOI:** 10.1136/bmj.a1682

**Published:** 2008-10-07

**Authors:** Linda Ganzini, Elizabeth R Goy, Steven K Dobscha

**Affiliations:** 1 Columbia Center for the Study of Chronic, Comorbid Mental and Physical Disorders, Health Services Research and Development, Portland Veterans Affairs Medical Center, PO Box 1034, Portland, OR 97239, USA; 2 Department of Psychiatry, Oregon Health and Science University

## Abstract

**Objective** To determine the prevalence of depression and anxiety in terminally ill patients pursuing aid in dying from physicians.

**Design** Cross sectional survey.

**Setting** State of Oregon, USA.

**Participants** 58 Oregonians, most terminally ill with cancer or amyotrophic lateral sclerosis, who had either requested aid in dying from a physician or contacted an aid in dying advocacy organisation.

**Main outcome measures** Diagnosis of depression or anxiety according to the hospital anxiety and depression scale and the structured clinical interview for the Diagnostic and Statistical Manual of Mental Disorders.

**Results** 15 study participants met “caseness” criteria for depression, and 13 met criteria for anxiety. 42 patients died by the end of the study; 18 received a prescription for a lethal drug under the Death with Dignity Act, and nine died by lethal ingestion. 15 participants who received a prescription for a lethal drug did not meet criteria for depression; three did. All three depressed participants died by legal ingestion within two months of the research interview.

**Conclusion** Although most terminally ill Oregonians who receive aid in dying do not have depressive disorders, the current practice of the Death with Dignity Act may fail to protect some patients whose choices are influenced by depression from receiving a prescription for a lethal drug.

## Introduction

In 1994 the voters of Oregon passed the Death with Dignity Act, which legalised the practice of physicians’ aid in dying for terminally ill patients. This law authorises a physician to prescribe a lethal dosage of drug, usually a short acting barbiturate, to a competent, requesting patient for the purposes of self administration.^1^ Several safeguards in the law ensure that patients are adult, competent, terminally ill, and choosing to end life voluntarily but not impulsively (box). Since enactment of the law in 1997, between one and two out of every thousand deaths in Oregon has been by lethal ingestion.^1^

**Legal requirements of the Oregon Death with Dignity Act^1^**

The attending physician who is responsible for care of the patient’s terminal illness must ensure that:

- The patient is aged 18 years or above
- The patient is a resident of the state of Oregon
- The patient has made one written and two oral requests separated by 15 days
- The patient understands the risks of aid in dying and the alternatives, including hospice and comfort care
- The patient is assessed by a consulting physician
- Information about the patient is reported to the Oregon Department of Human Services

The attending and consulting physicians must ensure that:

- The patient is capable of making and communicating healthcare decisions
- The decision is voluntary
- The patient has a terminal illness that would, within reasonable medical judgment, cause death within six months
- The patient is referred to a psychologist or psychiatrist if concern exists that the patient has a psychiatric disorder including depression that may impair judgment

Information from statistical reports are compiled by the Oregon Department of Human Services and published yearly^1^

The extent to which potentially treatable psychiatric disorders may influence patients’ decisions for aid in dying has been debated. For people at the end of life, depression, hopelessness, and psychosocial distress are among the strongest correlates of desire for hastened death.^2^ ^3^ ^4^ ^5^ ^6^ ^7^ ^8^ ^9^ Eighty per cent of patients with cancer who complete suicide have a mood disorder, and, in primary care populations, treatment of depression reduces suicidal ideation.^10^ ^11^ ^12^ ^13^ ^14^ The Death with Dignity Act requires that if the prescribing or consulting physician is concerned that the patient’s judgment is impaired by a mental disorder (such as depression) the patient must be referred to a psychiatrist or a psychologist. No drug can be prescribed until the mental health professional determines that the patient does not have a mental disorder causing impaired judgment.^1^ Physicians, hospice professionals, and family members of patients in Oregon who pursue aid in dying generally do not believe that depression influences choices for hastened death.^15^ ^16^ ^17^ In 2007 none of the people who died by lethal ingestion in Oregon had been evaluated by a psychiatrist or a psychologist.^1^ Healthcare professionals, however, often fail to recognise depression, particularly among medically ill patients.^18^ ^19^ ^20^ The goal of this study was to determine the prevalence and severity of psychological distress, including major depressive disorder, in Oregonians who request aid in dying.

## Methods

We used several sources to notify patients of the opportunity to participate in our study. Some potential participants had contacted Compassion and Choices of Oregon for information about accessing aid in dying. Compassion and Choices is an organisation that offers information and assistance to people who choose aid in dying in Oregon. In 2006 Compassion and Choices gave information to or attended the deaths of three quarters of patients who chose aid in dying.^21^ Other potential participants made an explicit request for aid in dying to a physician as outlined in the Death with Dignity Act. Ethics consultants and palliative medicine and oncology specialists in northwest Oregon invited these patients to participate. Patients referred from all sources contacted study personnel directly for more information about enrolling. All patients gave written informed consent to participate.

The study psychologist (ERG) administered all measures in the participant’s home. We used the MacArthur competence assessment tool to determine participants’ capacity to consent to research.^22^ Once enrolled, participants confirmed that they had expressed interest in obtaining aid in dying through Compassion and Choices or explicitly requested aid in dying from a physician. The protocol required exclusion of participants with cognitive impairment (23 or less on the Folstein McHugh mini-mental state examination or 7 or less on the short portable mental status questionnaire^23^ ^24^ ^25^). The psychologist administered the hospital anxiety and depression scale,^26^ which includes seven depression items and seven anxiety items, each rated on a 0-3 scale. For the purposes of identifying cases and consistent with expert recommendations, we identified participants with hospital anxiety and depression scale depression scores of 11 or greater as “depressed” and those with anxiety scores of 10 or greater as “anxious.”^27^ The psychologist administered the 20 item Beck hopelessness scale,^28^ a well validated measure of hopelessness, which is a predictor of suicidal ideation and suicide attempts.^29^ ^30^ We designated those with scores of 10 or greater as “hopeless.”

The psychologist completed the current mood disorder section of the structured clinical interview for American Psychiatric Association Diagnostic and Statistical Manual-IV axis I disorders (SCID-I), a standard research instrument for diagnosing mental disorders.^31^ Because knowing that the patient has requested aid in dying may influence diagnostic thresholds for depression, the SCID interview was audiotaped and the tapes were reviewed by a research psychiatrist (SKD) who did not know if the patient had requested aid in dying (19 audiotapes from terminally ill patients who had not requested aid in dying were randomly interspersed). Based on studies by Chochinov and colleagues,^32^ the severity of depressed mood or anhedonia needed to be at least moderate for the two weeks before the interview in order to reach the threshold for diagnosis. Chochinov et al also reported that when moderate thresholds were used for mood criteria, presence or absence of physical symptoms (such as weight loss or fatigue) no longer influenced categorisation of depression. Using an inclusive approach, we attributed all physical symptoms of depression to the diagnosis of depression, even if they might be a result of terminal disease. Thoughts of death or suicide and suicidal plans or attempts are criteria for major depressive disorder in the American Psychiatric Association Diagnostic and Statistical Manual.^31^ We attributed suicidal ideation to a diagnosis of depression only if the patient endorsed suicidal thoughts or plans aside from aid in dying. The final SCID diagnosis of major depressive disorder was reached by consensus if ERG and SKD disagreed. For the purposes of identifying cases of depression, we considered participants to be depressed if their SCID was positive or their hospital anxiety and depression scale depression score was 11 or greater.^27^

Participants rated their overall suffering in the two weeks before the interview on an 11 point scale with end points labelled 0=“I have not suffered” and 10=“I have suffered severely.”^33^ They rated their quality of life in the previous two weeks on an 11 point scale with 0=“Quality of life as good as it can be” and 10=“Terrible, very bad quality of life.” Participants rated their desire for death in the two weeks preceding the interview on an 11 point scale with end points labelled 0=“I desire to live as long as possible” and 10=“I have a strong desire to die soon.” Participants rated the influence of depression as a reason for requesting aid in dying on a scale on which 1=“depression not at all important in the decision to request a lethal prescription” and 5=“depression very important in the decision to request a lethal prescription.”

All participants diagnosed with major depressive disorder were notified of this result at the time of the study visit, and the study psychologist recommended treatment and offered to facilitate counselling. As is standard at our institution, a safety plan was developed so that all patients who seemed upset by participation in the study or were found to be imminently suicidal by means other than legalised assisted dying would be referred for an evaluation of mental health. Otherwise, participants were assured confidentiality in order to facilitate honest disclosure. We obtained information on outcomes—whether the study participant received a prescription of a lethal drug or died by lethal ingestion—six months or more after all other data collection was complete.

### Data analysis

We present data as frequencies and proportions for categorical items and as means and standard deviations for normally distributed continuous items. We used Student’s *t* test to compare means. All tests were two tailed and α was set at 0.05.

## Results

Of 178 Compassion and Choices clients notified of the opportunity to participate in the study, 12 were ineligible or deceased and 47 (28%) enrolled. The other 11 participants were referred from clinicians at other medical centres. No patients were excluded because of cognitive impairment or lack of capacity to consent to research. The mean age of the 58 patients requesting aid in dying was 66 (SD 12) years. Thirty one participants were women, 22 were married, and 21 were enrolled in a hospice at the time of the interview. The most common terminal diseases were cancer (n=44) and amyotrophic lateral sclerosis (n=7). At the time of the study interview 46 patients had explicitly requested aid in dying from a physician and 47 had contacted Compassion and Choices to obtain aid in dying.

Eight participants scored 11 or higher on the hospital anxiety and depression scale for depression, 13 scored 10 or greater on the anxiety subscale, and 11 scored 10 or higher on the Beck hopelessness scale. Twelve participants were diagnosed with depression by the SCID. Fifteen participants met our criteria for depression by being depressed on the SCID or having a hospital anxiety and depression scale depression score of 11 or higher. The mean desire to die among depressed participants was 5.7 (SD 3.0) on our 11 point scale. Seven of the depressed group did not attribute their pursuit of aid in dying to depression at all (score=1), but six felt that depression somewhat or strongly influenced their preference for hastened death (scores=3, 4, or 5). An offer to facilitate counselling was made to all depressed patients, but only one participant (patient C below) agreed.

Among the 42 participants who died by the end of the study, 18 received a prescription for a lethal drug and nine died by lethal ingestion. Among decedents, no significant differences existed between those who received a prescription for a lethal drug and those who did not on measures of psychosocial distress, except that those who received a prescription had (surprisingly) a lower desire to die and a trend toward lower hopelessness scores (table 1).

**Table 1 tbl1:** Comparison of deceased participants who received and did not receive prescription for lethal drug. Values are mean (SD) unless stated otherwise

Measure	Received prescription (n=18)	Did not receive prescription (n=22)	P value (*t* test
Hospital anxiety and depression scale—anxiety*	4.8 (3.2)	7.0 (5.1)	0.12
Hospital anxiety and depression scale—depression†	5.7 (3.4)	7.3 (4.4)	0.19
Hospital anxiety and depression scale—total‡	10.5 (5.4)	14.3 (8.6)	0.10
Beck hopelessness scale§	5.0 (3.0)	7.5 (5.4)	0.08
Desire to die¶	1.5 (2.6)	4.7 (3.7)	0.004
Suffering**	3.7 (2.7)	4.5 (2.9)	0.36
Quality of life††	4.0 (1.8)	5.1 (2.9)	0.13

*Scores range from 0 (no anxiety) to 21 (severe anxiety).^26^

†Scores range from 0 (no depression) to 21 (severe depression).^26^

‡Sum of anxiety and depression scales.^26^

§Scores range from 0 (not hopeless) to 20 (very hopeless).^28^

¶11 point scale: 0=I desire to live as long as possible; 10=I have a strong desire to die soon.

**11 point scale: 0=I have not suffered in the past two weeks; 10=I have suffered severely in the past two weeks.^33^

††11 point scale: 0=quality of life in past two weeks is as good as it can be; 10=quality of life in past two weeks is terrible, very bad.

Three of the 18 participants who received a prescription for a lethal drug met our criteria for depression on either the SCID or hospital anxiety and depression scale (table 2), and 15 did not. All three died by lethal ingestion in their home within two months of the interview. None had been evaluated by a mental health professional before participation in the research. Patient A, an elderly man with cancer who was receiving home hospice services, met “caseness” criteria on the hospital anxiety and depression scale with a depression score of 12, although his SCID result was negative. Patient B, a middle aged woman with cancer who was receiving home hospice services, was depressed by SCID criteria. She declined to complete the hopelessness scale because she had “trouble with the entire concept of hope.” She rated her desire to die and her suffering as quite high. Whether patients A and B received mental health evaluation or treatment after participation in the study is unknown. Patient C, an elderly woman with cancer, was depressed by SCID criteria. She received treatment for depression with a psychostimulant after completion of the survey, was subsequently enrolled in a hospice, and was documented by a psychiatrist to have a remission in her depression before her death. She received the prescription when she was depressed, and she reported that depression somewhat influenced her decision to pursue aid in dying.

**Table 2 tbl2:** Measures of psychological distress in depressed participants who received a physician’s aid in dying

Measure	Case A	Case B	Case C
SCID depression*	–	+	+
Hospital anxiety and depression scale—anxiety†	7	4	8
Hospital anxiety and depression scale—depression‡	12	10	9
Hospital anxiety and depression scale—total§	19	14	17
Beck hopelessness scale¶	9	NA	9
Desire to die**	6	8	5
Suffering††	4	8	5
How much depressed mood influenced decision‡‡	1	1	3

NA=not available; SCID=structured clinical interview for American Psychiatric Association Diagnostic and Statistical Manual-IV

*+ indicates major depressive disorder present; – indicates major depressive disorder absent.^31^

†Scores range from 0 (no anxiety) to 21 (severe anxiety).^26^

‡Scores range from 0 (no depression) to 21 (severe depression).^26^

§Sum of anxiety and depression subscales.^26^

¶Scores range from 0 (not hopeless) to 20 (very hopeless).^28^ Case B declined to complete this scale.

**11 point scale: 0=I desire to live as long as possible; 10=I have a strong desire to die soon.

††11 point scale: 0=I have not suffered in the past two weeks; 10=I have suffered severely in the past two weeks.^33^

‡‡1=depressed mood not at all important in decision to request prescription; 5=depressed mood very important in decision to request prescription.

## Discussion

Among patients who requested a physician’s aid in dying, one in four had clinical depression. However, more than three quarters of people who actually received prescriptions for lethal drugs did not have a depressive disorder. Our findings also indicate that the current practice of legalised aid in dying may allow some potentially ineligible patients to receive a prescription for a lethal drug; two of those who ultimately died by lethal ingestion had depression at the time they received a prescription for a lethal drug and died by ingesting the drug. A third patient was depressed at the time that she requested a physician’s aid in dying and probably received her prescription; she was successfully treated for her depression before she died by lethal ingestion.

### Strengths and limitations

Although many investigators have examined the degree to which depression is associated with a desire to die among terminally ill patients,^2^ ^3^ ^4^ ^5^ ^6^ ^7^ ^8^ ^9^ we believe that our study is the first to use standardised measures to examine the prevalence and severity of depression and anxiety in a group of patients who have actually requested and are potentially eligible to receive aid in dying.

The strengths of our study include a standard measure of depression (SCID) and a blinding system that controlled for the effect on the ultimate psychiatric diagnosis of the psychiatrist knowing that the patient had requested a physician’s aid in dying. The other measures of depression and anxiety are commonly used and well validated in terminally ill patients. Our study has several limitations. Use of an inclusive approach to categorise somatic symptoms, which, if present, were attributed to depression and not to terminal disease, carries the risk of inflating the prevalence of depressive disorder. In addition, only 28% of invited patients who requested aid in dying agreed to participate; uncertainty exists about the degree to which our data are generalisable to the entire population of patients who request physicians’ aid in dying. In a study of Oregon physicians who reported on 141 requests for aid in dying, only 36% of patients were in a hospice at the time of the request—similarly, only a third of our participants were yet enrolled in a hospice.^34^ In contrast, data collected by the Oregon Department of Human Services indicates that 86% of patients who die by prescription of a lethal drug are enrolled in a hospice before death.^1^ This suggests that most patients begin inquiring about a physician’s aid in dying before they enrol in a hospice. Our finding of a low proportion in hospice enrollees reflects this fact, rather than differences between our sample and all Oregonians who die by legal lethal ingestion.

The possibility remains that the three depressed patients who died by lethal ingestion satisfied the requirements of the Death with Dignity Act if the attending physician determined that depression was present but not influencing their judgment. The study participants themselves were divided in whether to attribute their interest in aid in dying to low mood. Although diagnosing depression can be relatively straightforward, determining its role in influencing decision making is more difficult, even by expert assessment. For example, in a study of 321 psychiatrists in Oregon only 6% were very confident that in a single evaluation they could adequately determine whether a psychiatric disorder was impairing the judgment of a patient requesting assisted suicide.^35^ In a study of 290 US forensic psychiatrists, 58% indicated that the presence of major depressive disorder should result in an automatic finding of incompetence for the purposes of obtaining assisted suicide.^36^ These data support that of the two components of the mental health assessment—presence of a disorder and determination of its influence—the greatest weight in determining eligibility for aid in dying should be on whether a relevant mental disorder such as depression can be diagnosed.

### Depression and desire for death

Other studies and surveys from Oregon have indicated that aid in dying among depressed patients is very rare. Physicians in Oregon who received requests for aid in dying from 143 patients after enactment of the Death with Dignity Act reported that 20% were depressed—a proportion comparable to what we found in this study. None of the depressed patients on whom they submitted information received a prescription for a lethal drug.^34^ Studies of healthcare providers, including hospice professionals, and family members in Oregon indicate that they believe that depression was rarely a factor influencing requests for a physician’s aid in dying.^15^ ^16^ ^17^ Our study suggests that in some cases depression is missed or overlooked.

In contrast, studies of interest in euthanasia from populations outside of Oregon suggest that depression and psychosocial distress are prominent among patients who endorse an interest in hastened death. For example, in a study of 200 terminally ill inpatients with cancer, the prevalence of depressive syndromes was 59% among patients with a serious and pervasive desire to die but only 8% among patients without such a desire.^6^ In a study of 988 terminally ill patients living in areas where physicians’ aid in dying was not legal, 10.6% reported seriously considering euthanasia or a physician’s aid in dying for themselves, and those with depressive symptoms were 25% more likely to endorse this.^9^ In a study of 98 patients admitted to a palliative care inpatient setting in the northeast United States, patients with major depression were four times more likely to have a high desire for hastened death.^8^

Whether findings from these patient groups can be extrapolated to patients who have actively requested legal physicians’ aid in dying has remained uncertain—although 17% of Oregonians are potentially interested in aid in dying, only 1-2% actually request it.^34^ ^37^ For example, in a study of 161 patients with cancer in Oregon who were longitudinally examined for interest in a physician’s aid in dying, 19 had a serious interest in aid in dying, fewer than half with a serious interest discussed aid in dying with their physician, two requested a prescription for a lethal drug, and none received one.^38^ This suggests that most terminally ill patients who declare interest in aid in dying do not actively pursue aid in dying under legalised conditions. As such, studies of preferences for aid in dying in these groups may misrepresent actual requesters of aid in dying. In contrast, our surveyed participants had taken active steps to pursue a physician’s aid in dying in one of the few jurisdictions where it is legal—all either explicitly requested aid in dying from a physician or contacted Compassion and Choices for information on the Oregon Death with Dignity Act. Before death, almost half had obtained a prescription for a lethal drug under the law.

### Conclusions

Our study suggests that most patients who request aid in dying do not have a depressive disorder. However, the current practice of the Death with Dignity Act in Oregon may not adequately protect all mentally ill patients, and increased vigilance and systematic examination for depression among patients who may access legalised aid in dying are needed. Tools for screening for depression such as those used in our study are easy to administer and may help to determine which patients need further evaluation by a mental health professional. Further study is needed to determine the effect of treatment of depression on the choice to hasten death.

**What is already known on this topic**

The state of Oregon legalised physicians’ aid in dying in 1997
Physicians, hospice professionals, and family members of patients who request a lethal drug do not believe that depression is an important reason why patients pursue aid in dying

**What this study adds**

Among terminally ill Oregonians who participated in our study and received a prescription for a lethal drug, one in six had clinical depression

## Notes

Ethical approval: The study was approved by the institutional review board of the Portland Veterans Affairs Medical Center and the participating medical centres.

Provenance and peer review: Not commissioned; externally peer reviewed.

## Notes

**Cite this as: ***BMJ* 2008;337:a1682

## References

[1] Oregon Department of Human Services. Death with Dignity Act. 2007. www.oregon.gov/DHS/ph/pas/index.shtml.

[2] Emanuel EJ. Depression, euthanasia, and improving end-of-life care. J Clin Oncol 2005;23:6456-8. 16116150 10.1200/JCO.2005.06.001

[3] Wilson KG, Chochinov HM, McPherson CJ, Skirko MG, Allard P, Chary S, et al. Desire for euthanasia or physician-assisted suicide in palliative cancer care. Health Psychol 2007;26:314-23. 17500618 10.1037/0278-6133.26.3.314

[4] Rosenfeld B, Breitbart W, Gibson C, Kramer M, Tomarken A, Nelson C, et al. Desire for hastened death among patients with advanced AIDS. Psychosomatics 2006;47:504-12. 17116952 10.1176/appi.psy.47.6.504

[5] Werth JL Jr. The relationships among clinical depression, suicide, and other actions that may hasten death. Behav Sci Law 2004;22:627-49. 15378592 10.1002/bsl.616

[6] Chochinov HM, Wilson KG, Enns M, Mowchun N, Lander S, Levitt M, et al. Desire for death in the terminally ill. Am J Psychiatry 1995;152:1185-91. 7625468 10.1176/ajp.152.8.1185

[7] Blank K, Robison J, Doherty E, Prigerson H, Duffy J, Schwartz HI. Life-sustaining treatment and assisted death choices in depressed older patients. J Am Geriatr Soc 2001;49:153-61. 11207869 10.1046/j.1532-5415.2001.49036.x

[8] Breitbart W, Rosenfeld B, Pessin H, Kaim M, Funesti-Esch J, Galietta M, et al. Depression, hopelessness, and desire for hastened death in terminally ill patients with cancer. JAMA 2000;284:2907-11. 11147988 10.1001/jama.284.22.2907

[9] Emanuel EJ, Fairclough DL, Emanuel LL. Attitudes and desires related to euthanasia and physician-assisted suicide among terminally ill patients and their caregivers. JAMA 2000;284:2460-8. 11074775 10.1001/jama.284.19.2460

[10] Henriksson MM, Isometsa ET, Hietanen PS, Aro HM, Lonnqvist JK. Mental disorders in cancer suicides. J Affect Disord 1995;36:11-20. 8988260 10.1016/0165-0327(95)00047-x

[11] Bruce ML, Ten Have TR, Reynolds CF 3rd, Katz II, Schulberg HC, Mulsant BH, et al. Reducing suicidal ideation and depressive symptoms in depressed older primary care patients: a randomized controlled trial. JAMA 2004;291:1081-91. 14996777 10.1001/jama.291.9.1081

[12] Unutzer J, Tang L, Oishi S, Katon W, Williams JW Jr, Hunkeler E, et al. Reducing suicidal ideation in depressed older primary care patients. J Am Geriatr Soc 2006;54:1550-6. 17038073 10.1111/j.1532-5415.2006.00882.x

[13] Conwell Y, Lyness JM, Duberstein P, Cox C, Seidlitz L, DiGiorgio A, et al. Completed suicide among older patients in primary care practices: a controlled study. J Am Geriatr Soc 2000;48:23-9. 10642017 10.1111/j.1532-5415.2000.tb03024.x

[14] Conwell Y, Duberstein PR, Caine ED. Risk factors for suicide in later life. Biol Psychiatry 2002;52:193-204. 12182926 10.1016/s0006-3223(02)01347-1

[15] Ganzini L, Harvath TA, Jackson A, Goy ER, Miller LL, Delorit MA. Experiences of Oregon nurses and social workers with hospice patients who requested assistance with suicide. N Engl J Med 2002;347:582-8. 12192019 10.1056/NEJMsa020562

[16] Carlson B, Simopolous N, Goy ER, Jackson A, Ganzini L. Oregon hospice chaplains’ experiences with patients requesting physician-assisted suicide. J Palliat Med 2005;8:1160-6. 16351529 10.1089/jpm.2005.8.1160

[17] Ganzini L, Goy ER, Dobscha SK. Why Oregon patients request assisted death: family members’ views. J Gen Intern Med 2008;23:154-7. 18080719 10.1007/s11606-007-0476-x PMC2265314

[18] Passik SD, Dugan W, McDonald MV, Rosenfeld B, Theobald DE, Edgerton S. Oncologists’ recognition of depression in their patients with cancer. J Clin Oncol 1998;16:1594-600. 9552071 10.1200/JCO.1998.16.4.1594

[19] Simon GE, VonKorff M. Recognition, management, and outcomes of depression in primary care. Arch Fam Med 1995;4:99-105. 7842160 10.1001/archfami.4.2.99

[20] Perez-Stable EJ, Miranda J, Munoz RF, Ying YW. Depression in medical outpatients: underrecognition and misdiagnosis. Arch Intern Med 1990;150:1083-8. 2184790 10.1001/archinte.1990.00390170113024

[21] Compassion and Choices of Oregon. 2007. www.compassionoforegon.org.

[22] Appelbaum PS, Grisso T. MacArthur competence assessment tool for clinical research (MacCAT-CR). Sarasota, FL: Professional Resource Press, 2001.

[23] Folstein MF, Folstein SE, McHugh PR. “Mini-mental state”: a practical method for grading the cognitive state of patients for the clinician. J Psychiatr Res 1975;12:189-98. 1202204 10.1016/0022-3956(75)90026-6

[24] Smyer MA, Hofland BF, Jonas EA. Validity study of the short portable mental status questionnaire for the elderly. J Am Geriatr Soc 1979;27:263-9. 447988 10.1111/j.1532-5415.1979.tb06128.x

[25] Pfeiffer E. A short portable mental status questionnaire for the assessment of organic brain deficit in elderly patients. J Am Geriatr Soc 1975;23:433-41. 1159263 10.1111/j.1532-5415.1975.tb00927.x

[26] Zigmond AS, Snaith RP. The hospital anxiety and depression scale. Acta Psychiatr Scand 1983;67:361-70. 6880820 10.1111/j.1600-0447.1983.tb09716.x

[27] Hotopf M, Chidgey J, Addington-Hall J, Ly KL. Depression in advanced disease: a systematic review. Part 1: prevalence and case finding. Palliat Med 2002;16:81-97. 11969152 10.1191/02169216302pm507oa

[28] Beck AT, Weissman A, Lester D, Trexler L. The measurement of pessimism: the hopelessness scale. J Consult Clin Psychol 1974;42:861-5. 4436473 10.1037/h0037562

[29] Beck A, Brown G, Berchick R, Stewart B, Steer R. Relationship between hopelessness and ultimate suicide: a replication with psychiatric outpatients. Am J Psychiatry 1990;147:190-5. 2278535 10.1176/ajp.147.2.190

[30] Minkoff K, Bergman E, Beck A, Beck R. Hopelessness, depression, and attempted suicide. Am J Psychiatry 1973;130:455-9. 4691303 10.1176/ajp.130.4.455

[31] First MB, Spitzer RL, Gibbon M, Williams JBW. User’s guide for the structured clinical interview for DSM-IV axis I disorders: clinician version. New York: Biometrics Research Department, 1997.

[32] Chochinov HM, Wilson KG, Enns M, Lander S. Prevalence of depression in the terminally ill: effects of diagnostic criteria and symptom threshold judgments. Am J Psychiatry 1994;151:537-40. 7511875 10.1176/ajp.151.4.537

[33] Ganzini L, Johnston WS, Hoffman WF. Correlates of suffering in amyotrophic lateral sclerosis. Neurology 1999;52:1434-40. 10227631 10.1212/wnl.52.7.1434

[34] Ganzini L, Nelson HD, Schmidt TA, Kraemer DF, Delorit MA, Lee MA. Physicians’ experiences with the Oregon Death with Dignity Act. N Engl J Med 2000;342:557-63. 10684915 10.1056/NEJM200002243420806

[35] Ganzini L, Fenn DS, Lee MA, Heintz RT, Bloom JD. Attitudes of Oregon psychiatrists toward physician-assisted suicide. Am J Psychiatry 1996;153:1469-75. 8890683 10.1176/ajp.153.11.1469

[36] Ganzini L, Leong GB, Fenn DS, Silva JA, Weinstock R. Evaluation of competence to consent to assisted suicide: views of forensic psychiatrists. Am J Psychiatry 2000;157:595-600. 10739419 10.1176/appi.ajp.157.4.595

[37] Tolle SW, Tilden VP, Drach LL, Fromme EK, Perrin NA, Hedberg K. Characteristics and proportion of dying Oregonians who personally consider physician-assisted suicide. J Clin Ethics 2004;15:111-8. 15481162

[38] Ganzini L, Beer TM, Brouns M, Mori M, Hsieh YC. Interest in physician-assisted suicide among Oregon cancer patients. J Clin Ethics 2006;17:27-38. 16689111

